# Health literacy levels of British adults: a cross-sectional survey using two domains of the Health Literacy Questionnaire (HLQ)

**DOI:** 10.1186/s12889-020-09727-w

**Published:** 2020-11-30

**Authors:** Rebecca M. Simpson, Emma Knowles, Alicia O’Cathain

**Affiliations:** School of Health and Related Research, University of Sheffield, Sheffield, UK

**Keywords:** Health literacy, Health literacy questionnaire (HLQ), Health information, Population survey, Ability to engage, Understanding information, Communication

## Abstract

**Background:**

A person’s health literacy determines whether they are able to make appropriate health decisions and are able to follow treatment instructions. This is important because low health literacy is associated with mortality and extra costs to the healthcare system. Our aim was to describe the health literacy levels of British adults using a nationally representative population survey, and show how health literacy levels vary by population characteristics.

**Methods:**

A population based cross-sectional survey including questions from two domains from the Health Literacy Questionnaire™: 1) Understanding health information well enough to know what to do, and 2) Ability to actively engage with health care providers. Both domains are made up of 5 Likert style questions with 5 levels ranging from ‘cannot do or always difficult’ (1) to ‘always easy’ (5). The survey was conducted by NatCen in Britain (2018) as part of the annual British Social Attitudes survey. We used weighted descriptive analyses and regression to explore the relationship between population characteristics and health literacy. Weighted analyses were used to ensure the sample was representative of the British population.

**Results:**

A total of 2309 responded to the questionnaire. The mean score for ‘understanding information’ was 3.98 (95% CI: 3.94, 4.02) and for ‘ability to engage’ was 3.83 (95% CI: 3.80, 3.87), where 5 is the highest score. 19.4% had some level of difficulty reading and understanding written health information, and 23.2% discussing health concerns with health care providers. The adjusted logistic regression for ‘understanding information’ showed that those with lower health literacy were more likely to be in the most socially deprived quintile (OR 2.500 95% CI: 1.180, 5.296), have a limiting health condition or disability (OR 4.326 95% CI: 2.494, 7.704), and have no educational qualifications (OR 7.588 95% CI: 3.305, 17.422). This was similar for the ‘ability to engage’ domain.

**Conclusions:**

This study described the distribution of health literacy levels for the British population in 2018. Interventions to improve health literacy will best be targeted at those with lower levels of education, those living in the most deprived areas, and those with a limiting health condition or disability.

## Background

A person’s health literacy determines whether they are able to make appropriate health decisions and are able to follow treatment instructions [1, 2]. Health literacy is based on a person’s capability to understand, read, use and obtain health care information. Health literacy is important because low health literacy is associated with mortality [3], extra costs to the health care system [4] and lower levels of medication adherence [5].

Different instruments exist to measure health literacy. Some focus on objective measurement such as the Newest Vital Sign [6] and the Test of Functional Health Literacy in Adults [7] whereas others focus on a person’s subjective assessment [8–12]. The Health Literacy Questionnaire™ (HLQ) is a measure based on a person’s subjective assessment. It was developed and validated in Australia [8] and has been used to measure health literacy in different countries [13, 14]. It consists of nine domains of health literacy: feeling understood and supported by healthcare providers, having sufficient information to manage health, actively managing health, social support for health, appraisal of health information, ability to actively engage with healthcare providers, navigating the healthcare system, ability to find good health information, and understanding health information enough to know what to do [8].

As part of a wider study focusing on decision-making when seeking emergency and urgent care, we measured health literacy within a population survey. We selected the HLQ™ as the most appropriate instrument to use because it is well-validated and easy to complete within a survey. We selected two of the nine domains that focus on understanding health information (‘understanding information’) and ability to actively engage with health professionals (‘ability to engage’), because these are important for decision-making around seeking healthcare. A high score in the ‘understanding information’ domain indicates that a person feels capable of understanding written and numerical information about their health, including being able to complete forms relating to their treatment [8]. A high score in the ‘ability to engage’ domain indicates that the person feels able to be proactive when it comes to their health and feel in control in their relationships with health care professionals [8].

A number of studies have measured health literacy, using various tools, within different countries, investigating the level of health literacy and the characteristics affecting health literacy [15–17]. A study in Denmark in 2013 [15] found the largest differences in health literacy scores occurred by income and educational attainment, with those in lower income and education groups having lower health literacy. The Danish study also found that men had lower scores than women for the ‘understanding information’ domain of the HLQ™. A study in Australia in 2013–2014 found that the lowest health literacy scores occurred in those with lower education, those born overseas, and those who were not English speaking at home [16]. Differences were also seen for age, gender, chronic conditions and living arrangements [16]. A study in a single city in the UK in 2013 found that those in older age groups and those with lower education were more likely to have limited health literacy [17]. Deprivation was also found to impact on health literacy, with those in the most socially deprived groups more likely to have lower health literacy scores [17].

The aim of our study was to describe the health literacy levels of British adults using a nationally representative population survey and show how health literacy levels vary by population characteristics.

## Methods

### Study population and data collection

A population based cross-sectional survey was conducted in Britain. The survey was undertaken by NatCen Social Research who conduct an annual survey researching British social attitudes [18]. The survey is designed to be a representative sample of adults (over 18 years old) in Britain. They do this by using a three stage design. They start by selecting 395 postcode sectors with a probability that is proportional to the number of addresses in that sector. They then select 26 addresses within each sector; this produces 10,270 addresses. Finally, the interviewers call at each address and randomly select one adult over 18 to interview. The survey consisted of face to face administration of the questionnaire by an interviewer for most of the questions and a self-complete questionnaire for a small proportion of the questions. The questionnaire consisted of around 300 items administered to 4000 people. We bought a 60-item module for our wider study, based on a representative sample of 3000 people. A license was obtained to use two health literacy domains (‘understanding information’ and ‘ability to engage’) from the HLQ™, consisting of 10 items. These were asked within the self-completed questionnaire.

The survey was undertaken in the summer of 2018. The response rate to the whole British Social Attitudes Survey was 42%. Of those who completed the face to face interview, the response rate for the self-competed questionnaire for our module was 79% (2309) [18]. See Table 6 in the appendix for the characteristic breakdown between the interview administered and self-completed samples.

### Population characteristics

A number of characteristics were collected as part of the survey: age, sex, living in household with children under 5, geographical region, educational attainment, living alone, ethnicity, income, whether they had visited a GP in the past 12 months and whether they were living with limiting long term conditions. NatCen provided deprivation scores using the Index of Multiple Deprivation (IMD) quintiles, and urban rural status with the dataset based on postcode of the respondent.

### Statistical analysis

All analyses were completed using SPSS version 25. NatCen Social Research produced weights to address sample bias due to both selection probabilities and non-response, and to ensure the sample matched the population profile in terms of age, sex and geographical region. Separate weights were produced for interviewer-administered questions and self-completed questions due to differential response rates. The ‘complex samples function’ in SPSS was used for weighting the analysis, based on the self-completed weights.

The scores for each of the two health literacy domains were calculated from the 10 health literacy questions using the instrument’s scoring rules. Each question had 5 responses: 1 = Cannot do or always difficult, 2 = Usually difficult, 3 = Sometimes difficult, 4 = Usually easy and 5 = Always easy. For both the ‘understanding information’ and ‘ability to engage’ domains, the score range is 1–5 and the overall score for each domain is an average score across all the questions from that domain. Missing data was imputed using the Expectation Maximisation algorithm. As both the scales were made up of 5 questions, missing data was only imputed if there were no more than two questions missing within a domain. If there were more the two questions missing, then a score was not calculated for that individual in that domain.

Analysis included frequencies and descriptive statistics of the individual health literacy items and the two domains scores, both overall and within the population characteristics described earlier. Generalised linear models within the SPSS complex sample function were used to compare means of both domains by each population characteristic (i.e. t-test and ANOVA).

To measure the relationship between the population characteristics and the two domains, linear regression was used. Univariable (unadjusted) linear regression was used for each characteristic variable and multivariable (adjusted) linear regression was used with all the characteristic variables to model both domains. All population characteristics were chosen a priori based on previous relevant literature [15, 16].

A binary variable was created to determine whether a person was in a ‘lower health literacy’ group for each domain because this offers more meaningful results. There is no recommended cut-off point to indicate low health literacy. We chose a cut off of ≤3 for a domain because scores of 1 to 3 on each item indicate a level of difficulty (‘cannot do or always difficult’, ‘usually difficult’ or ‘sometimes difficult’). Similarly to the linear regression analysis, univariable (unadjusted) logistic regression was used for each characteristic variable and multivariable (adjusted) logistic regression was used with all the characteristic variables to model both domains. The Odds Ratios (OR) presented show the odds of being in the ‘lower health literacy’ group compared to being in the ‘higher health literacy’ group.

### Ethics approval

The NatCen Research Ethics Committee (REC) approved the British Social Attitudes survey (reference number P12598).

## Results

### Description of sample

The unweighted and weighted sample is presented in Table 1. The mean age of respondents was 54, ranging between 18 and 99. For ethnicity, 91% of the respondents were white and weighting changed this to 85%, increasing the weight of Black Asian Minority Ethnic (BAME) people in the analysis.

**Table 1:** Description of sample *N* = 2309

		Unweighted Count	Unweighted %	Weighted %	Unweighted missing
**Sex**	Male	974	42.2%	47.8%	0
	Female	1335	57.8%	52.2%	
**Age**	18–24	133	5.8%	10.7%	5
	25–34	291	12.6%	17.1%	
	35–44	348	15.1%	15.9%	
	45–54	382	16.6%	17.8%	
	55–64	414	18.0%	15.4%	
	65–74	424	18.4%	13.8%	
	75+	312	13.5%	9.3%	
**Number of children under 5 years old living in household**	0	2075	90.3%	87.9%	11
	1+	223	9.7%	12.1%	
**Region**	North	377	16.3%	16.0%	0
	Midlands	617	26.7%	24.2%	
	South	785	34.0%	32.1%	
	London	219	9.5%	13.8%	
	Wales	107	4.6%	5.4%	
	Scotland	204	8.8%	8.5%	
**IMD Quintile**	1 (Most deprived)	418	18.1%	21.5%	0
	2	405	17.5%	19.2%	
	3	448	19.4%	18.2%	
	4	528	22.9%	20.5%	
	5 (Least deprived)	510	22.1%	20.6%	
**Urban Rural**	Urban	1750	75.8%	78.6%	0
	Rural	559	24.2%	21.4%	
**Long Term Condition**	No long term health condition or disability	1376	59.8%	63.1%	7
	Non-limiting health condition or disability	499	21.7%	20.2%	
	Limiting health condition or disability	427	18.5%	16.7%	
**Education**	Degree or equivalent	640	28.2%	28.0%	36
	A level or equivalent	616	27.1%	26.9%	
	GCSE or equivalent	590	26.0%	26.9%	
	No Qualification	427	18.8%	18.2%	
**Live Alone**	Alone	702	30.4%	17.3%	0
	Not alone	1607	69.6%	82.7%	
**Visited GP in the last 12 months**	In last 12 months	1924	83.3%	83.1%	0
	More than 12 Months/never	385	16.7%	16.9%	
**Household income**	Less than £1200 p.m	462	21.9%	18.6%	202
	£1200–2200 p.m	470	22.3%	21.3%	
	£2201–3700 p.m	433	20.6%	21.3%	
	£3701 or more p.m	456	21.6%	23.5%	
	Refused information	286	13.6%	15.3%	
**Ethnicity**	White	2098	90.9%	85.2%	0
	Black Asian and Minority Ethnic (BAME)	211	9.1%	14.8%	

*p.m per month*

*IMD* Index of Multiple Deprivation, *GCSE* General Certificate of Secondary Education

### Description of health literacy levels

The weighted distribution of responses to each item is presented in Table 2. Most of the population responded ‘always easy’ or ‘usually easy’ to all 10 items but around one in five had some level of difficulty. For example, 19.4% had some level of difficulty reading and understanding written health information (item 3U), and 23.2% discussing health concerns with health care providers (item 2A).

**Table 2:** Weighted response to each question in the ‘understanding information’ and ‘ability to engage’ domains

Items^**a**^	Cannot do or always difficult	Usually difficult	Sometimes difficult	Usually easy	Always easy	Low Health Literacy^**b**^
**UNDERSTANDING INFORMATION**						
**Confidently fill medical forms in the correct way (1U)**	1.5%	4.7%	15.4%	58.0%	20.4%	21.6%
**Accurately follow instructions from … (2U)**	0.9%	1.8%	12.5%	62.8%	21.9%	15.2%
**Read and understand written health information (3U)**	1.4%	3.4%	14.6%	56.2%	24.4%	19.4%
**Read and understand all the information on medication labels (4U)**	1.4%	3.3%	14.9%	57.2%	23.2%	19.6%
**Understand what healthcare providers are asking you to do (5U)**	0.9%	2.3%	13.3%	63.4%	20.1%	16.5%
**ABILITY TO ENGAGE**						
**Make sure that healthcare providers understand your problems properly (1A)**	1.6%	5.2%	28.3%	54.9%	10.2%	35.1%
**Feel able to discuss your health concerns with a healthcare provider (2A)**	1.1%	4.1%	18.0%	60.7%	16.1%	23.2%
**Have good discussions about your health with doctors (3A)**	1.9%	5.1%	18.7%	55.9%	18.4%	25.7%
**Discuss things with healthcare providers until you understand all you need to (4A)**	1.2%	3.6%	20.1%	56.2%	18.9%	24.9%
**Ask healthcare providers questions to get the health information … (5A)**	0.9%	4.2%	17.6%	58.4%	19.0%	22.7%

^*a*^*Some of the HLQ™ items have been truncated. HLQ™ is protected by copyright and cannot be used without permission of the authors. Full copy of the items is available at* hlq@deakin.edu.au*or* globalhealthandequity@swin.edu.au

^*b*^*Low health literacy defined as ‘Cannot do …’, ‘Usually difficult’ and ‘Sometimes difficult’*

The mean score for the ‘understanding information’ domain was 3.98 (95% CI: 3.94, 4.02). The mean score for the ‘ability to engage’ domain was 3.83 (95% CI: 3.80, 3.87). Both domains had a modal score of 4, with scores ranging from 1 to 5.

### Health literacy by population characteristics

Table 3 presents the itemised proportions of those who selected some level of difficulty (‘cannot do or always difficult’, ‘usually difficult’ or ‘sometimes difficult’) for each domain and the weighted mean score for each domain by the various subgroups.

**Table 3:** Weighted means of the ‘understanding information’ and ‘ability to engage’ domains by population characteristics

Characteristics		Understanding Information						Ability to Engage					
		Weighted % reporting difficulty^**a**^ across items					Mean (95% CI) N	Weighted % reporting difficulty^**a**^ across items					Mean (95% CI) N
		1U	2U	3U	4U	5U		1A	2A	3A	4A	5A	
**Sex**	Male	25.4	17.3	21.2	21.2	19.8	3.91 (3.85, 3.96) 956	34.9	23.3	25.4	26.0	24.1	3.81 (3.76, 3.86) 955
	Female	18.1	13.3	17.8	18.1	13.5	4.04 (4.00, 4.09) 1313	34.9	23.0	26.1	23.9	21.3	3.85 (3.80, 3.89) 1314
**Age**	18–24	24.9	15.9	22.9	25.1	23.7	3.95 (3.82, 4.07) 130	42.4	26.4	32.3	33.2	31.0	3.73 (3.60, 3.86) 130
	25–34	21.4	15.4	19.3	20.3	20.5	3.96 (3.87, 4.06) 290	39.8	28.2	30.6	27.7	22.6	3.76 (3.68, 3.85) 289
	35–44	20.0	19.7	19.1	19.2	16.4	4.00 (3.90, 4.10) 345	36.2	25.5	30.3	26.5	25.4	3.83 (3.74, 3.92) 345
	45–54	17.2	11.2	18.0	15.9	13.3	4.02 (3.95, 4.10) 377	34.5	23.5	23.6	22.1	17.8	3.87 (3.79, 3.94) 377
	55–64	24.3	14.8	18.2	16.9	16.2	3.98 (3.90, 4.07) 408	30.5	21.9	22.2	21.9	21.7	3.85 (3.76, 3.93) 408
	65–74	21.9	15.2	17.5	19.2	10.9	3.98 (3.91, 4.05) 416	28.6	15.6	18.6	19.7	19.1	3.91 (3.83, 3.98) 416
	75+	25.5	14.3	23.3	24.5	15.8	3.90 (3.83, 3.98) 298	33.1	18.4	22.1	25.6	24.4	3.86 (3.78, 3.95) 299
**Number of children aged 0-4 yrs**	0	22.1	14.7	18.8	19.7	15.9	3.98 (3.95, 4.02) 2035	34.2	22.8	24.8	24.2	22.2	3.84 (3.80, 3.88) 2035
	1+	15.5	15.8	21.2	16.3	18.3	3.97 (3.87, 4.08) 223	38.9	24.4	30.8	28.1	23.6	3.78 (3.69, 3.87) 223
**Region**	North	17.6	14.8	19.7	19.7	15.2	4.02 (3.95, 4.10) 369	34.3	20.3	21.8	21.0	19.8	3.87 (3.79, 3.94) 370
	Midlands	25.0	17.0	22.1	20.9	17.7	3.93 (3.85, 4.00) 609	40.6	25.2	27.4	25.6	22.9	3.80 (3.73, 3.87) 609
	South	17.6	11.0	16.1	17.5	12.9	4.03 (3.98,4.07) 774	30.7	21.4	26.8	23.9	22.6	3.85 (3.80, 3.91) 774
	London	25.2	23.9	20.9	22.6	25.0	3.93 (3.78, 4.08) 215	32.8	26.0	26.4	32.4	24.2	3.82 (3.69, 3.94) 214
	Wales	26.6	11.4	15.9	17.5	16.3	4.03 (3.87, 4.20) 103	37.5	26.5	21.4	22.1	25.1	3.83 (3.65, 4.02) 103
	Scotland	26.2	15.6	22.3	20.0	15.3	3.91 (3.80, 4.02) 199	37.9	22.5	23.0	23.6	23.1	3.79 (3.67, 3.92) 199
**IMD Quintile**	1 (Most)	34.0	26.2	30.4	28.0	27.1	3.78 (3.69, 3.87) 410	45.6	34.9	32.9	31.9	30.1	3.67 (3.58, 3.75) 411
	2	24.3	17.7	21.1	22.0	19.9	3.91 (3.82, 3.99) 392	37.1	23.6	31.0	30.6	25.6	3.75 (3.68, 3.83) 392
	3	18.7	11.8	17.0	19.4	14.0	4.03 (3.97, 4.10) 443	34.7	18.8	24.1	24.2	21.6	3.87 (3.80, 3.94) 443
	4	17.1	11.3	14.8	12.4	11.8	4.08 (4.02, 4.14) 520	28.8	19.6	21.4	21.6	19.6	3.92 (3.86, 3.97) 519
	5 (Least)	13.3	8.6	12.8	15.8	9.2	4.10 (4.05, 4.16) 504	28.4	17.8	19.2	16.1	16.2	3.95 (3.89, 4.01) 504
**Urban or Rural**	Urban	21.9	16.3	19.6	20.0	17.7	3.98 (3.93, 4.02) 1721	36.3	24.0	26.5	26.3	23.4	3.82 (3.78, 3.86) 1721
	Rural	20.9	11.1	18.7	18.0	12.3	3.98 (3.91,4.05) 548	30.3	20.3	22.7	20.0	19.9	3.87 (3.80, 3.94) 548
**Long term condition or disability**	No long term health condition or disability	17.3	12.6	16.7	16.9	14.3	4.03 (3.99, 4.08) 1351	31.9	20.6	22.0	22.0	19.6	3.87 (3.84, 3.92) 1350
	Non-limiting health condition or disability	21.3	14.6	19.1	20.4	17.2	4.00 (3.93, 4.07) 493	30.5	21.7	26.6	24.4	23.3	3.85 (3.79, 3.92) 493
	Limiting health condition or disability	38.8	25.7	30.0	29.3	24.2	3.75 (3.65, 3.84) 419	52.4	34.7	39.5	36.8	34.0	3.59 (3.49, 3.69) 420
**Education**	Degree or equivalent	8.6	5.8	7.5	11.5	9.0	4.20 (4.15, 4.26) 634	24.4	17.0	19.8	19.2	15.0	3.98 (3.92, 4.05) 633
	A level or equivalent	17.6	14.0	16.6	16.0	15.3	4.02 (3.96, 4.08) 607	34.3	20.5	25.8	22.7	21.5	3.84 (3.78, 3.90) 607
	GCSE or equivalent	25.6	14.7	21.1	22.3	16.2	3.94 (3.87, 4.00) 583	39.1	24.7	27.6	25.8	22.8	3.79 (3.73, 3.86) 583
	No Qualification	42.5	32.1	38.8	33.9	30.6	3.64 (3.55, 3.73) 411	45.3	33.8	31.5	35.1	35.0	3.66 (3.57, 3.75) 412
**Live Alone**	Alone	29.0	20.2	25.9	24.5	20.6	3.83 (3.75, 3.91) 688	37.7	26.8	28.2	28.6	26.3	3.72 (3.65, 3.80) 688
	Not alone	20.2	14.2	18.0	18.6	15.6	4.01 (3.97, 4.05) 1581	34.4	22.4	25.2	24.1	21.9	3.85 (3.81, 3.89) 1581
**Visited GP in the last 12 months**	In last 12 months	22.0	15.5	19.8	19.9	16.0	3.97 (3.93, 4.01) 1900	34.7	22.8	26.1	24.7	23.1	3.83 (3.79, 3.87) 1900
	More than 12 Months/never	20.2	13.9	17.3	18.1	18.4	4.00 (3.92, 4.08) 369	36.4	24.9	24.0	25.6	20.4	3.83 (3.75, 3.91) 369
**Household income**	Less than £1200 p.m	33.5	23.7	28.5	27.9	22.5	3.78 (3.68, 3.87) 452	44.4	30.4	31.7	30.5	30.1	3.67 (3.58, 3.76) 452
	£1200–2200 p.m	22.8	11.9	18.7	22.0	14.6	4.00 (3.93, 4.07) 461	34.8	22.6	23.7	24.2	22.3	3.84 (3.77, 3.92) 461
	£2201–3700 p.m	13.7	12.5	14.6	15.7	15.0	4.06 (3.99, 4.14) 430	34.6	22.1	26.0	23.3	19.3	3.88 (3.81, 3.96) 429
	£3701 or more p.m	11.7	7.5	11.0	9.5	9.0	4.15 (4.10, 4.21) 452	27.6	16.1	22.0	17.5	17.1	3.94 (3.88, 4.00) 452
	Refused information	25.9	23.1	25.8	23.4	22.1	3.90 (3.78, 4.01) 277	33.6	25.1	27.6	30.4	26.2	3.80 (3.71, 3.90) 277
**Ethnicity**	White	20.6	12.6	17.8	18.1	14.0	4.00 (3.97, 4.03) 2064	33.1	20.7	25.5	23.5	21.8	3.84 (3.81, 3.88) 2064
	BAME	27.7	30.2	28.6	27.6	30.9	3.85 (3.70, 4.00) 205	46.2	37.2	27.7	32.5	27.3	3.75 (3.62, 3.88) 205

^*a*^ *Those who selected ‘Cannot do or always difficult’, ‘Usually difficult’ or ‘Sometimes difficult’*

The following subgroups had generally higher proportions of individuals selecting some level of difficulty over the five questions for both ‘understanding information’ and ‘ability to engage’: most socially deprived quintile, with a limiting health condition or disability, people who live alone, lower household incomes and BAME. This is reflected in the mean scores, with the following subgroups having lower scores for both domains: men, most socially deprived quintile, with a limiting health condition or disability, lower levels of education, people who live alone, lower household incomes and BAME. For example, for one of the items in the ‘understanding information’ domain, 34% of people living in the most socially deprived communities expressed some level of difficulty compared with 13% in the most affluent quintile.

### Regression analysis

Table 4 presents the unadjusted and adjusted linear regression results for both the ‘understanding information’ and ‘ability to engage’ domains for each of the subgroups of interest, based on the mean of each domain.

**Table 4:** Unadjusted and adjusted linear regression models for ‘understanding information’ and ‘ability to engage’ by subgroups (significant results are in bold and have a * at *p* < 0.05)

Variable (Reference Category)		Understanding Information				Ability to Engage			
		Unadjusted		Adjusted^**a**^		Unadjusted		Adjusted^**a**^	
		***β***	(95% CI)	***β***	(95% CI)	***β***	(95% CI)	***β***	(95% CI)
**Sex (Female)**	Male	− 0.136*	**(− 0.201, − 0.070)**	− 0.130*	**(− 0.197, − 0.063)**	− 0.040	(− 0.107, 0.026)	− 0.046	(− 0.114, 0.023)
**Age (18–24)**	25–34 35–44 45–54 55–64 65–74 75+	0.018 0.054 0.076 0.038 0.033 − 0.044	(− 0.134, 0.170) (− 0.106, 0.214) (− 0.070, 0.221) (− 0.115, 0.191) (− 0.111, 0.178) (− 0.205, 0.117)	0.090 − 0.055 0.024 0.017 0.016 0.057	(− 0.079, 0.258) (− 0.204, 0.095) (− 0.130, 0.177) (− 0.132, 0.166) (− 0.131, 0.162) (− 0.091, 0.205)	0.032 0.099 0.136 0.117 0.178* 0.131	(− 0.130, 0.194) (− 0.061, 0.259) (− 0.009, 0.281) (− 0.037, 0.271) **(0.030, 0.326)** (− 0.032, 0.294)	0.249* − 0.028 0.083 0.111 0.118 0.196*	**(0.053, 0.445)** (− 0.203, 0.146) (− 0.080, 0.246) (− 0.050, 0.271) (− 0.044, 0.279) **(0.033, 0.359)**
**Children Under 5 (0)**	1+	− 0.011	(− 0.115, 0.094)	− 0.056	(− 0.176, 0.063)	− 0.059	(− 0.157, 0.039)	− 0.049	(− 0.164, 0.066)
**Region (Scotland)**	North Midlands South London Wales	0.111 0.015 0.115 0.017 0.121	(− 0.021, 0.242) (− 0.118, 0.148) (− 0.005, 0.234) (− 0.169, 0.203) (− 0.077, 0.320)	0.109 − 0.035 0.022 0.034 0.087	(− 0.030, 0.248) (− 0.163, 0.092) (− 0.094, 0.137) (− 0.139, 0.206) (− 0.094, 0.268)	0.074 0.005 0.058 0.024 0.040	(− 0.069, 0.218) (− 0.137, 0.148) (− 0.078, 0.194) (− 0.153, 0.201) (− 0.183, 0.263)	0.086 − 0.011 − 0.007 0.018 0.000	(− 0.076, 0.247) (− 0.167, 0.146) (− 0.157, 0.144) (− 0.173, 0.210) (− 0.215, 0.214)
**IMD (5 Least deprived)**	1 (Most deprived) 2 3 4	− 0.325* − 0.196* − 0.070 − 0.022	**(− 0.429, − 0.220)** **(− 0.299, − 0.092)** (− 0.153, 0.013) (− 0.096, 0.051)	− 0.168* − 0.079 0.003 0.043	**(− 0.282, − 0.053)** (− 0.184, 0.025) (− 0.078, 0.085) (− 0.032, 0.119)	− 0.282* − 0.195* − 0.075 − 0.033	**(− 0.385, − 0.179)** **(− 0.294, − 0.096)** (− 0.164, 0.013) (− 0.111, 0.045)	− 0.155* − 0.091 − 0.002 0.009	**(− 0.272, − 0.037)** (− 0.201, 0.020) (− 0.100, 0.096) (− 0.081, 0.100)
**Urban vs Rural (Rural)**	Urban	− 0.004	(− 0.086, 0.077)	0.094*	**(0.012, 0.176)**	− 0.049	(− 0.129, 0.030)	0.021	(− 0.063, 0.105)
**Long Term Condition (No long term health condition or disability)**	Non-limiting health condition or disability Limiting health condition or disability	− 0.035 − 0.287*	(− 0.118, 0.048) **(− 0.388, − 0.186)**	− 0.031 − 0.172*	(− 0.118, 0.055) **(− 0.285, − 0.059)**	− 0.034 − 0.298*	(− 0.113, 0.046) **(− 0.401, − 0.195)**	− 0.072 − 0.254*	(− 0.161, 0.017) **(− 0.375, − 0.132)**
**Education (Degree or equivalent)**	A level or equivalent GCSE or equivalent No Qualification	−0.181* − 0.264* − 0.565*	**(− 0.255, − 0.106)** **(− 0.347, − 0.182)** **(− 0.675, − 0.455)**	− 0.157* − 0.220* − 0.444*	**(− 0.243, − 0.072)** **(− 0.310, − 0.129)** **(− 0.567, − 0.322)**	− 0.146* − 0.190* − 0.326*	**(− 0.232, − 0.059)** **(− 0.280, − 0.101)** **(− 0.442, − 0.210)**	− 0.136* − 0.176* − 0.281*	**(− 0.230, − 0.042)** **(− 0.271, − 0.080)** **(− 0.414, − 0.148)**
**Live Alone (Alone)**	Not Alone	0.183*	**(0.098, 0.269)**	0.125*	**(0.043, 0.207)**	0.129*	**(0.047, 0.210)**	0.112*	**(0.030, 0.194)**
**Visit GP (In last 12 months)**	More than 12 Months/never	0.030	(− 0.054, 0.114)	0.021	(− 0.072, 0.115)	0.004	(− 0.084, 0.091)	− 0.041	(− 0.137, 0.054)
**Income (Less than £1200 p.m)**	£1200–2200 p.m. £2201–3700 p.m. £3701 or more p.m. Refused information	0.224* 0.290* 0.377* 0.121	**(0.105, 0.343)** **(0.173, 0.406)** **(0.267, 0.487)** (− 0.026, 0.269)	− 0.017 0.087 0.112 0.088	(− 0.159, 0.125) (− 0.028, 0.202) (− 0.006, 0.230) (− 0.032, 0.208)	0.172* 0.212* 0.267* 0.129	**(0.058, 0.287)** **(0.096, 0.328)** **(0.156, 0.377)** (− 0.004, 0.262)	0.009 0.074 0.077 0.055	(− 0.126, 0.144) (− 0.044, 0.192) (− 0.047, 0.200) (− 0.073, 0.184)
**Ethnicity (White)**	BAME	− 0.151	(− 0.303, 0.001)	0.096	(− 0.054, 0.246)	− 0.093	(− 0.225, 0.039)	0.025	(− 0.117, 0.166)

*Adjusted sample size: ‘understanding information’: n = 2026, ‘ability to engage’: n = 2025.*

^a^Model adjusted for all subgroups variables in the table

The adjusted regression results for the ‘understanding information’ domain suggest that those with lower health literacy scored were males (−0.130 95% CI: −0.197, −0.063) compared to females, those in the most socially deprived quintile (−0.168 95% CI: −0.282, −0.053) compared to those in the highest deprivation quintile, those who have a limiting health condition or disability (−0.172 95% CI: −0.285, −0.059) compared to those who do not have one, and all education levels compared to those with a degree, ranging from −0.157 to −0.444. Finally, those who do not live alone have a higher health literacy score (0.125 95% CI: 0.043, 0.207) compared to those who do live alone.

The adjusted regression for the ‘ability to engage’ domain suggests those with a lower health literacy score were the most deprived (−0.155 95% CI: −0.272, −0.037) compared to the least deprived group, those who have a limiting health condition or disability (−0.254 95% CI: −0.375, −0.132) compared to those who do not have one, and all education levels when compared to those with a degree, ranging from −0.136 to −0.281. The results suggest that those not living alone have a higher health literacy score (0.112 95% CI: 0.030, 0.194) compared to those who do live alone.

Table 5 shows the results of a comparison of the characteristics of the proportion of the population with lower health literacy levels (likely to have expressed some level of difficulty). It displays the unadjusted and adjusted logistic regression results for both the ‘understanding information’ and ‘ability to engage’ domains for each of the subgroups of interest.

**Table 5:** Unadjusted and adjusted logistic regression models for ‘understanding information’ and ‘ability to engage’ by subgroups (significant results are in bold and have a * at *p* < 0.05)

Variable (Reference Category)		Understanding Information				Ability to Engage			
		Unadjusted		Adjusted^**a**^		Unadjusted		Adjusted^**a**^	
		***β***	(95% CI)	***β***	(95% CI)	***β***	(95% CI)	***β***	(95% CI)
**Sex (Female)**	Male	1.305	(0.900, 1.890)	1.217	(0.792,1.869)	1.105	(0.820, 1.489)	1.131	(0.807, 1.585)
**Age (18–24)**	25–34 35–44 45–54 55–64 65–74 75+	1.510 1.277 0.856 0.899 0.770 1.288	(0.670, 3.401) (0.493, 3.309) (0.353, 2.075) (0.365, 2.213) (0.302, 1.967) (0.514, 3.225)	1.727 1.086 0.910 0.749 0.511 0.549	(0.631, 4.730) (0.363, 3.243) (0.301, 2.752) (0.237, 2.365) (0.153, 1.707) (0.157, 1.919)	0.866 0.806 0.587 0.775 0.461* 0.588	(0.432, 1.739) (0.407, 1.598) (0.312, 1.104) (0.417, 1.441) **(0.226, 0.941)** (0.299, 1.153)	0.975 0.688 0.507 0.579 0.323* 0.251*	(0.434, 2.192) (0.328, 1.444) (0.241, 1.067) (0.271, 1.236) **(0.138, 0.756)** **(0.095, 0.669)**
**Children Under 5 (0)**	1+	1.265	(0.702, 2.277)	1.078	(0.478, 2.436)	0.892	(0.542, 1.468)	0.700	(0.371, 1.322)
**Region (Scotland)**	North Midlands South London Wales	0.961 1.340 0.659 1.928 0.590	(0.458, 2.017) (0.666, 2.694) (0.329, 1.320) (0.835, 4.450) (0.149, 2.346)	0.893 1.702 0.898 1.225 0.761	(0.388, 2.058) (0.796, 3.640) (0.429, 1.881) (0.490, 3.058) (0.253, 2.291)	0.771 0.949 0.620 1.054 0.977	(0.423, 1.406) (0.558, 1.617) (0.367, 1.047) (0.561, 1.979) (0.428, 2.228)	0.699 0.925 0.725 0.950 1.263	(0.386, 1.267) (0.522, 1.641) (0.423, 1.244) (0.464, 1.945) (0.576, 2.769)
**IMD (5 Least deprived)**	1 (Most deprived) 2 3 4	6.024* 3.655* 2.245* 1.289	**(3.229, 11.239)** **(1.884, 7.090)** **(1.164, 4.328)** (0.622, 2.673)	2.500* 1.559 1.185 0.715	**(1.180, 5.296)** (0.750, 3.242) (0.567, 2.477) (0.328, 1.559)	3.136* 2.079* 1.417 1.379	**(2.009, 4.895)** **(1.296, 3.335)** (0.875, 2.296) (0.876, 2.171)	2.020* 1.411 1.041 1.301	**(1.177, 3.467)** (0.824, 2.419) (0.592, 1.829) (0.786, 2.153)
**Urban vs Rural (Rural)**	Urban	1.389	(0.890, 2.168)	0.625	(0.353, 1.108)	1.249	(0.890, 1.751)	0.875	(0.606, 1.262)
**Long Term Condition (No long term health condition or disability)**	Non-limiting health condition or disability Limiting health condition or disability	1.250 3.587*	(0.705, 2.213) **(2.218, 5.800)**	1.840* 4.326*	**(1.000, 3.385)** **(2.494, 7.504)**	1.522* 3.031*	**(1.052, 2.200)** **(2.077, 4.424)**	1.882* 3.102*	**(1.284, 2.758)** **(1.939, 4.963)**
**Education (Degree or equivalent)**	A level or equivalent GCSE or equivalent No Qualification	1.470 2.534* 8.662*	(0.762, 2.835) **(1.427, 4.498)** **(4.648, 16.142)**	1.624 2.537* 7.588*	(0.711, 3.710) **(1.178, 5.463)** **(3.305, 17.422)**	1.287 1.678* 2.855*	(0.831, 1.995) **(1.125, 2.504)** **(1.796, 4.536)**	1.36 1.716* 2.973*	(0.834, 2.217) **(1.063, 2.769)** **(1.557, 5.674)**
**Live Alone (Alone)**	Not Alone	0.510*	**(0.351, 0.741)**	0.602*	**(0.368, 0.986)**	0.702*	**(0.519, 0.952)**	0.756	(0.525, 1.088)
**Visit GP (In last 12 months)**	More than 12 Months/never	0.988	(0.574, 1.702)	1.077	(0.561, 2.069)	0.986	(0.653, 1.489)	1.129	(0.716, 1.781)
**Income (Less than £1200 p.m)**	£1200–2200 p.m. £2201–3700 p.m. £3701 or more p.m. Refused information	0.467* 0.283* 0.151* 0.604	**(0.277, 0.788)** **(0.149, 0.539)** **(0.075, 0.306)** (0.332, 1.100)	0.974 0.622 0.789 1.015	(0.279, 1.388) (0.324, 1.921) (0.324, 1.921) (0.544, 1.744)	**0.573** 0.549* 0.428* 0.605*	**(0.370, 0.886)** **(0.351, 0.859)** **(0.273, 0.671)** **(0.378, 0.968)**	0.812 0.966 1.027 0.872	(0.489, 1.348) (0.559, 1.671) (0.574, 1.836) (0.509, 1.496)
**Ethnicity (White)**	BAME	3.546*	**(2.179, 5.780)**	3.472*	**(1.721, 6.993)**	1.680*	**(1.120, 2.540)**	1.399	(0.775, 2.525)

*Odd ratios represent the odds of being in the ‘lower literacy’ group compared to the ‘higher literacy group’. Lower literacy is a score ≤ 3 on the final domain score*

*Adjusted sample size: ‘understanding information’: n = 2026, ‘ability to engage’: n = 2025*

^a^Model adjusted for all subgroups variables in the table

The adjusted logistic regression results for the ‘understanding information’ domain suggest that those who were more likely to be in the ‘lower health literacy’ group were those most socially deprived (OR 2.500 95% CI: 1.180, 5.296) compared to the least deprived group, both those with a non-limiting health condition or disability (OR 1.840 95% CI: 1.000, 3.385) and a limiting health condition or disability compared to those who do not have one (OR 4.326 95% CI: 2.494, 7.704), those with lower levels of education (OR ranging from 2.537 to 7.588) when compared to those with a degree, and those from BAME communities (OR 3.472 95% CI: 1.721, 6.993) when compared to white population. Those not living alone were less likely to be in the ‘lower health literacy’ group (OR 0.602 95% CI: 0.363, 0.986) when compared to those who do live alone.

Similarly, the adjusted logistic regression results for the ‘ability to engage’ domain suggest that those who were more likely to be in the ‘lower health literacy’ group were those most socially deprived (OR 2.020 95% CI: 1.177, 3.467) compared to the least deprived group, both those with a non-limiting health condition or disability (OR 1.882 95% CI: 1.284, 2.758) and a limiting health condition or disability compared to those who do not have one (OR 3.102 95% CI: 1.939, 4.963) and those with lower levels of education (OR ranging from 1.716 to 2.973) when compared to those with a degree.

## Discussion

The health literacy levels of the British population are described here. The overall mean score for the ‘understanding information’ domain was 3.98 (95% CI: 3.94, 4.02) and the overall mean score for the ‘ability to engage’ domain was 3.83 (95% CI: 3.80, 3.87). 19.4% had some level of difficulty reading and understanding written health information, and 23.2% discussing health concerns with health care providers. The adjusted logistic regression for the ‘understanding information’ domain showed that those with lower health literacy were more likely to be in the most socially deprived quintile, have a limiting health condition or disability, have no educational qualifications and be from Black Asian and Minority Ethnic communities. This was similar for the ‘ability to engage’ domain with the exception of the finding about BAME.

The Australian Bureau of Statistics (ABS) conducted a national health survey in 2018 exploring health literacy levels using all domains of the HLQ™. For the ‘ability to engage’ domain, 11% of the population reported some degree of difficulty to engage, whereas the remaining percentage found it easy. Similarly, for the ‘understanding information’ domain, 7% of the population reported difficultly when trying to understand information [19]. This study reported smaller percentages of lower health literacy than we found in our results. It is not easy to make comparisons between countries as there is no validated questionnaire for inter country comparison. When comparing results between different countries caution needs to be taken as the context and social attitudes are different which could affect how respondents score health literacy questions. Another similar study, conducted in Denmark in 2013, found that 12.8% had some level of difficulty reading and understanding written health information, and 14.5% had some level of difficulty discussing health concerns with health care providers [15]. Again, these percentages are smaller than our study results. It is important to note, that even though the same questionnaire was used in the Denmark study, they used a four point scale instead of five.

Our results about the characteristics of lower health literacy were similar to those found in other countries and using different measures of health literacy, including objective measures. A study conducted in a city in England found that those living in the most deprived areas were twice as likely to have low health literacy compared to those in the least deprived area [17]. A number of studies have shown that education impacts on health literacy, with lower education associated with lower health literacy levels [15–17]. Living alone has been found to be associated with lower health literacy in Denmark [15], but not in an Australian study [16]. The Australian study investigated the effect of having four or more chronic conditions on a person’s health literacy; they found an association but this was not statistically significant for the ‘ability to engage’ and ‘understanding information’ domains [16]. Another study conducted in Denmark concluded that those with chronic conditions found it more difficult to understand health information and engage with healthcare providers [13]. All of these studies also used the Health Literacy Questionnaire™.

There were some differences between our study and others. In the Danish study, similarly to our study they found that males had lower health literacy for the ‘understanding information’ domain but in contrast they found that males had higher scores for the ‘ability to engage’ domain [15].

### Strengths and limitations

This study had two main strengths. First, the design and analysis of the survey means the results are likely to be representative of the British population. Second, the HLQ™ is a well validated questionnaire [8, 20, 21]. The study also had four potential limitations. First, as participation in the survey was voluntary, some of those asked may have declined due to having lower levels of English language and literacy. Alongside this, the health literacy questions were part of the self-complete questionnaire so for the same reasons, people with poorer literacy may have decided not to return the questionnaire. In the appendix we show a comparison between the interview administered sample and the self-complete sample, showing a small under representation of people from socially deprived communities completing the health literacy items Table 6. This suggests that our results are likely to overestimate health literacy levels in Britain. Second, we opted to include only two of the nine health literacy domains in the HLQ™, so only addressed some aspects of health literacy. Third, some of the ORs are large due to small sample sizes in some categories. Fourth, the analysis was conducted using the complex samples function within SPSS to allow for the sample weighting. A limitation of this function is that non-parametric methods cannot be used and the data were slightly skewed. However, given the sample size and the robustness of parametric methods, we do not believe that this was a problem in practice.

### Implications

Given the association between low health literacy and mortality, lower medication adherence and extra health care costs, variation in health literacy levels are of concern. This study has identified groups to target with interventions including socially deprived communities, those with low education, those with limiting health condition or disability, and those living alone. The survey reported here has also offered a national baseline for any national initiative to improve health literacy in the future.

## Conclusion

This study has described the distribution of health literacy levels for ‘understanding information’ and ‘ability to engage’ with health professionals for the British population in 2018. Interventions to improve health literacy will best be targeted at those with lower levels of education, those living in the most deprived areas, those with a limiting health condition or disability and those who live alone.

## Data Availability

NatCen make the data from the British Social Attitudes survey available using their own data sharing processes.

## Appendix

**Table 6:** Differences between interview administered and self-complete sample

Characteristic		Interview administered Sample Count ***N*** = 2906	%	Missing	Self-complete Sample Count ***N*** = 2309	%	Missing
**Sex**	Male Female	1257 1649	43.3 56.7	0	974 1335	42.2 57.8	0
**Age**	18–24 25–34 35–44 45–54 55–64 65–74 75+	169 384 467 469 508 499 405	5.8 13.2 16.1 16.2 17.5 17.2 14.0	5	133 291 348 382 414 424 312	5.8 12.6 15.1 16.6 18.0 18.4 13.5	5
**Number of children under 5 years old living in household**	0 1+	2591 300	89.6 10.4	15	2075 223	90.3 9.7	11
**Region**	North Midlands South London Wales Scotland	474 794 957 285 132 264	16.3 27.3 32.9 9.8 4.5 9.1	0	377 617 785 219 107 204	16.3 26.7 34.0 9.5 4.6 8.8	0
**IMD Quintile**	1 (Most deprived) 2 3 4 5 (Least deprived)	576 545 536 638 611	19.8 18.8 18.4 22.0 21.0	0	418 405 448 528 510	18.1 17.5 19.4 22.9 22.1	0
**Urban Rural**	Urban Rural	2241 665	77.1 22.9	0	1750 559	75.8 24.2	0
**Long Term Condition**	No long term health condition or disability Non-limiting health condition or disability Limiting health condition or disability	1766 586 541	61.0 20.3 18.7	13	1376 499 427	59.8 21.7 18.5	7
**Education**	Degree or equivalent A level or equivalent GCSE or equivalent No Qualification	763 760 750 582	26.7 26.6 26.3 20.4	51	640 616 590 427	28.2 27.1 26.0 18.8	36
**Live Alone**	Alone Not alone	913 1993	31.4 68.6	0	702 1607	30.4 69.6	0
**Visited GP in the last 12 months**	In last 12 months More than 12 Months/never	2386 519	82.1 17.9	1	1924 385	83.3 16.7	0
**Household income**	Less than £1200 p.m. £1200–2200 p.m. £2201–3700 p.m. £3701 or more p.m. Refused information	568 577 506 528 439	21.7 22.0 19.3 20.2 16.8	288	462 470 433 456 286	21.9 22.3 20.6 21.6 13.6	202
**Ethnicity**	White BAME	2572 334	88.5 11.5	0	2098 211	90.9 9.1	0

## Abbreviations

HLQ™: Health literacy questionnaire™
BAME: Black Asian Minority Ethnic
IMD: Index of Multiple Deprivation
GCSE: General Certificate of Secondary Education
CI: Confidence interval
